# Spatial analysis and soft computational modeling for hazard assessment of potential toxic elements in potable groundwater

**DOI:** 10.1038/s41598-024-76147-w

**Published:** 2024-10-26

**Authors:** R. S. Aswal, Mukesh Prasad, Jaswinder Singh, Hakam Singh, Utpal Shrivastava, Manoj Wadhwa, Om Prakash Pandey, Johnbosco C. Egbueri

**Affiliations:** 1 Department of Environmental Sciences, H.N.B. Garhwal University, Badshahi Thaul Campus, Tehri Garhwal, 249 199 India; 2 Department of Medical Physics, Himalayan Institute of Medical Sciences, Swami Rama Himalayan University, Jolly Grant, Dehradun, 248 016 India; 3 Department of Computer Science and Engineering, Guru Jambheshwar University of Science and Technology, Hisar, Haryana 125 001 India; 4 Chitkara University School of Engineering and Technology, Chitkara University, Himachal Pradesh, 174 103 India; 5 Department of Computer Science and Engineering, University Institute of Engineering, Chandigarh University, Mohali, 140413 Punjab India; 6 Physical Science and Engineering Division, King Abdullah University of Science and Technology (KAUST), Thuwal, 23955-6900 Saudi Arabia; 7 Department of Geology, Chukwuemeka Odumegwu Ojukwu University, Uli, Nigeria

**Keywords:** Health risk assessment, Machine learning, Pollution indices, Potential toxic elements, Environmental sciences, Natural hazards

## Abstract

Swiftly increasing population and industrial developments of urban areas has accelerated the worsening of the water quality in recent years. Groundwater samples from different locations of the Doon valley, Garhwal Himalaya were analyzed to measure concentrations of six potential toxic elements (PTEs) viz. chromium (Cr), nickel (Ni), arsenic (As), molybdenum (Mo), cadmium (Cd), and lead (Pb) using Inductively Coupled Plasma Mass Spectrometer (ICP-MS) with the aim to study the spatial distribution and associated hazards. In addition, machine learning algorithms have been used for prediction of water quality and identification of influencing PTEs. The results inferred that the mean values (in the units of µg L^−1^) of analyzed PTEs were observed in the order of Mo (1.066) > Ni (0.744) > Pb (0.337) > As (0.186) > Cr (0.180) > Cd (0.026). The levels and computed risks of PTEs were found below the safe limits. The radial basis function neural network (RBF-NN) algorithms showed high level of accuracy in the predictions of heavy metal pollution index (HPI), heavy metal evaluation index (HEI), non-carcinogenic (N-CR) and carcinogenic (CR) parameters with determination coefficient values ranged from 0.912 to 0.976. However, the modified heavy metal pollution index (m-HPI) and contamination index (CI) predictions showed comparatively lower coefficient values as 0.753 and 0.657, respectively. The multilayer perceptron neural network (MLP-NN) demonstrated fluctuation in precision with determination coefficient between 0.167 and 0.954 for the prediction of computed indices (HPI, HEI, CI, m-HPI). In contrast, the proficiency in forecasting of non-carcinogenic and carcinogenic hazards for both sub-groups showcased coefficient values ranged from 0.887 to 0.995. As compared to each other, the radial basis function (RBF) model indicated closer alignments between predicted and actual values for pollution indices, while multilayer perceptron (MLP) model portrayed greater precision in prediction of health risk indices.

## Introduction

The easy and affordable accessibility to clean potable water is one of the fundamental human rights. Water quality is significantly connected with preservation of ecosystems, economic growth, and social development of nations^1–3^. Unfortunately, the quality of several groundwater supplies around the world has been hindered due to rapid economic developments, urbanization, natural disasters, and increasing human activities^4–7^. This degradation has led to the excessive release of potentially toxic elements (PTEs) into water resources of many countries, resulting in a gradual decline in groundwater quantity^8,9^. Water quality degradation manifests in the occurrence of a wide variety of pollutants, including high levels of PTEs, various cations and anions, particularly nitrogen compounds such as nitrate^10–12^, as well as bacterial contamination. Among these pollutants, PTEs are of particular concern even at minute levels^13,14^. While PTEs naturally occur in geological bodies of the earth and frequently get released to groundwater sources through natural processes^15,16^, their levels can be exacerbated by human activities.

The excess of PTEs in water makes it unsuitable for potable and other domestic applications^17^. Additionally, industrial processes could potentially contribute to water quality issues if not properly managed. These may include chemical synthesis operations, such as those producing formic acid^18^, and energy extraction methods like underground coal gasification^19^, which can affect surrounding geological structures and potentially impact groundwater quality. It is essential to assess health hazards of toxicants in groundwater of poor quality for monitoring its impact on the human beings. The human exposure to various heavy metals (HMs) is also due to their bioaccumulation in different sources of foods^20,21^. Thus, individuals not only consume HMs directly through water, but from plant and other food sources also^22,23^. Various mathematical and statistical methods have been applied by researchers to deliver better insights of the water quality and related human health risk^24–31^. These methods are effective and useful in assessing the water quality for drinking purpose. The United States Environmental Protection Agency’s (USEPA) risk assessment approach has garnered significant attention from the scientific community in recent years, both domestically and globally^32,33^.

So far, there have been limited studies conducted in the Garhwal Himalayan Doon Valley, mostly focusing on the application of traditional descriptive and statistical methods to analyze physicochemical characteristics, ionic composition, and HMs concentrations in water quality indicators^34–39^. Despite an urban region, the groundwater sources of the Doon valley region are still less explored in view of the human health risks assessment. Further studies are required to be focused on HMs concentrations in groundwater sources of this region and HM exposure through oral and dermal pathways. Therefore, data intelligent numerical (indexical) and soft computing (machine learning) models have been combined for predictive modelling of groundwater quality in this region with the aim (i) to analyze and appraise the spatial variation in physicochemical characteristics and HMs concentration; (ii) to categorize the groundwater sources and their fitness for drinking purpose using different pollution indices; (iii) to appraise non-carcinogenic (N-CR) and carcinogenic health hazards of HMs in groundwater linked with oral and dermal exposure pathways using human health risk assessment (HHRA) model of the USEPA for both adults and children; (iv) to predict the HPI, m-HPI, HEI, CI, hazard index (HI), and Cancer risk (CR) for both age sub-groups using radial basis function (RBF) and multilayer perceptron neural networks (MLP-NNs) models; (v) to identify the most sensitive HMs influencing to the RBF and MLP-NNs models and (vi) to compare the performances of developed RBF and MLP-NNs models.

## Materials and methods

### Study area

**Figure 1:**
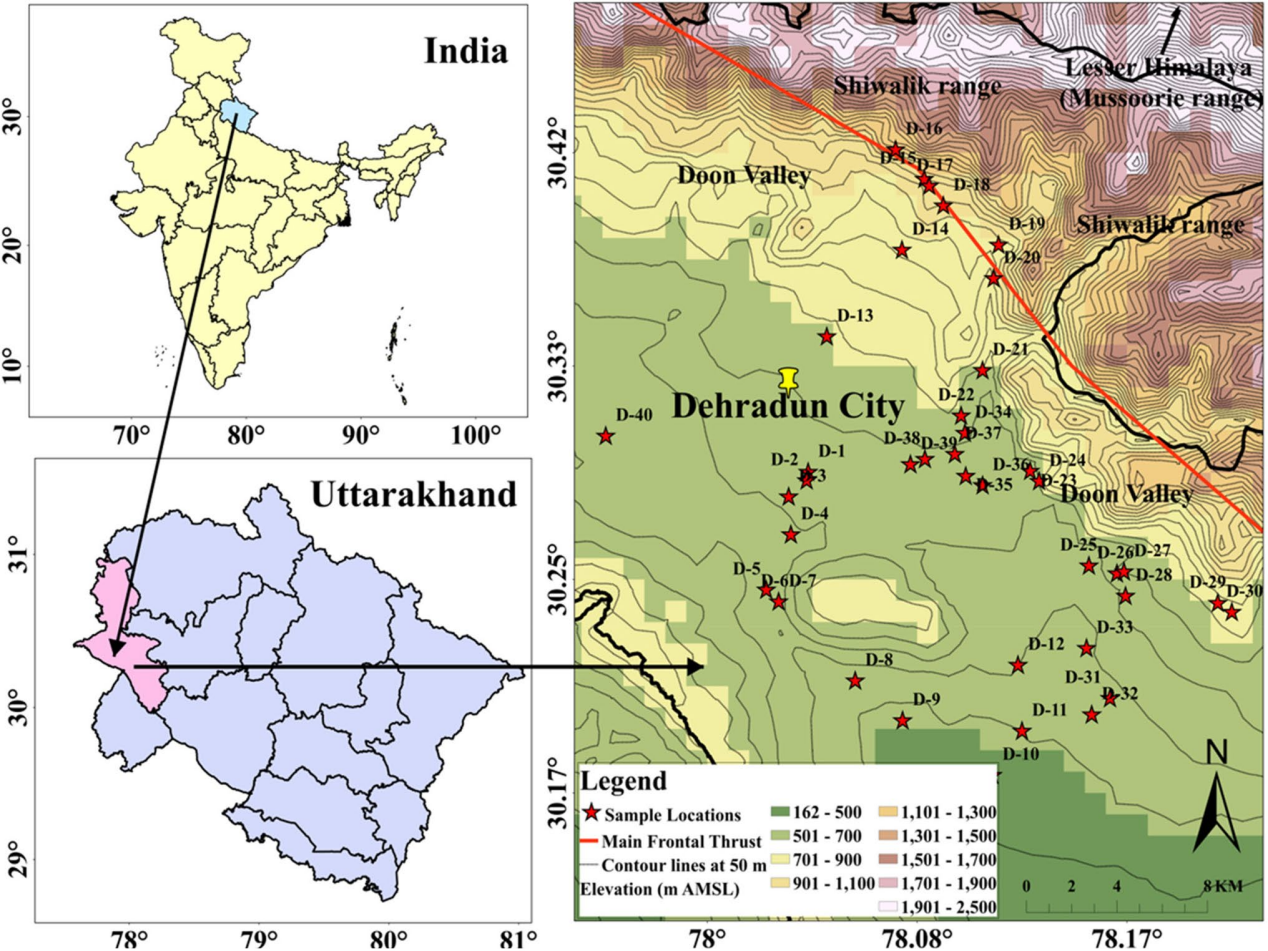
Sampling map of the study area (prepared with ArcGIS, version 10.7.1, URL: https://www.esri.com/en-us/arcgis/products/arcgis-desktop/resources) in Doon valley of Garhwal Himalaya, India.

The potable groundwater samples (borewells and natural springs) were collected from the Doon valley situated in north west corner of the Uttarakhand State in India. Figure 1 displays the study area’s sampling map. The Shivalik Hills region encompasses the inter-mountain Doon valley positioned between the Mussorrie range of the lesser Himalaya to the north and the Shivalik hills range to the south^40^.

The Mussorrie range and Shivalik Hills range are separated by a major structure called Main Boundary Thrust (MBT). Doon valley is further divided into three geological zones namely Lesser Himalayan Zone, Central Valley Zone and Shiwalik Zone^41^. Covering an expanse of 3,088 square kilometers, the Dehradun district comprises 17 towns and 764 villages. According to the 2011 census, the overall population of the entire Dehradun district is approximately 1.7 million, with nearly 0.8 million females and 0.9 million males. The entire district is drained by Ganga, Yamuna and a few of their tributaries. Song and Suswa are two main tributaries of the Ganges river system. The majority of the study area’s yearly rain (roughly 2000 mm), falls between June and September. The rainiest months of the season are July and August among them.

### Collection and preparation of water samples

The selection of sampling locations (*N* = 40) for water sample collection was strategically done to encompass the widest possible area. The public typically relies on these groundwater sources to fulfill their needs for drinking, preparing food, and other household chores. In the current study, the collection, preservation, transportation, and analysis of samples were as per the standard protocols^42^. A comprehensive description of the sample collection and preparation procedures is elaborated in our recent article^31,43^.

### Chemical analysis, quality control (QC) and quality assurance (QA) in laboratory

Concentrations of six PTEs i.e., chromium (Cr), nickel (Ni), arsenic (As), molybdenum (Mo), cadmium (Cd) and lead (Pb) in the groundwater samples were measured using an Agilent 8900 Triple Quad Inductively Coupled Plasma Mass Spectrometer (ICP-MS). The labwares used during sample preparation and analysis were pre-cleaned using diluted (2% v/v) analytical-grade nitric acid and Milli-Q ultra-pure water. The ICP-MS was calibrated for Cr, Ni, As, Mo, Cd, and Pb using 10, 50, and 250 ppb concentration calibration solutions (prepared using a Certipur^®^ ICP multi-element standard solution XVI; Merck KGaA, Germany) and a blank (2% v/v analytical-grade nitric acid). A standard reference material NIST SRM 1643f was analyzed with the samples for quality control and quality assurance. All water samples, calibration standard solutions, quality control standard solution (NIST SRM 1643f), and blank were doped with internal standard Rh (Merck KGaA, Germany). The internal standard was used to monitor the signal stability of the ICP-MS and also to correct any drifting in it. The analysis was performed in the He mode and concentrations of PTEs were quantified against a 4-point calibration curve having *R* ≥ 0.999. Three replicates of NIST SRM 1643f were analyzed before and after each batch of six samples and yielded an accuracy and precision of better than 10% for each batch. Further details regarding analytical procedure, quality control and quality assurance are discussed elsewhere^31^.

### Pollution assessment of groundwater

The pollution assessment of groundwater aquifers with respect to HMs was done by estimating the water quality indices (WQIs). The WQIs are composite measurements that include the potential danger to human health from the presence of significant pollutants in groundwater. It provides an overview of the water quality status of representative water body. In this study, four WQIs viz. heavy metal pollution index (HPI), modified-heavy metal pollution index (m-HPI), Heavy metal evaluation index (HEI), and contamination index (CI) were employed to assess quality of the analyzed groundwater samples. The detailed description of these pollution indices is described along with the parameters in our earlier article^31^.

### Risks estimation

The human health risk assessment model of the USEPA was employed to assess non-carcinogenic and carcinogenic health hazards via direct consumption and skin penetration for children (average 10 years) and adults (average 70 years)^44^.

The chronic daily intake (CDI) for direct ingestion (*CDI*_*ing*_) and dermal contact (*CDI*_*der*_) were calculated for an individual PTE using following Eqs. (1) and (2)^45^. The input parameters used in estimating health risks are shown in Table 1.

1 $$\boldsymbol\:{\mathbf{CDI}}_{\mathbf{ing}\boldsymbol-\mathbf{nc}}=\frac{\mathbf{EC}\boldsymbol\times\boldsymbol\:\mathbf{DWI}\boldsymbol\times\boldsymbol\:\mathbf{EF}\boldsymbol\times\boldsymbol\:\mathbf{EP}}{\mathbf{LE}\boldsymbol\times\boldsymbol\:\mathbf{BW}}$$

2 $$\boldsymbol\:{\mathbf{CDI}}_{\mathbf d\mathbf e\mathbf r\boldsymbol-\mathbf n\mathbf c}=\frac{\mathbf E\mathbf C\times\mathbf S\mathbf A\times{\mathbf K}_p\times\mathbf E\mathbf T\times\mathbf E\mathbf F\times\mathbf E\mathbf P\times\mathbf C\mathbf F}{\mathbf L\mathbf E\boldsymbol\times\mathbf B\mathbf W}$$

**Table 1:** Different variables used in health risk assessment.

EC	Concentration of the analyzed element
DWI	Daily water intake (3.45 L per day^−1^ for adults, for children 2 L day^−1^)
EF	Exposure frequency (365 days year^−1^)
EP	Exposure period (70 years for adults, 10 years for children)
LE	Life expectancy (25,550 days for adults, 3250 days for children)
BW	Body weight (73 kg for adults, 32.7 kg for children)
K_p_	Dermal permeability coefficient (cm h^−1^)
SA	Exposed skin area (18000 cm^2^ for adults, 6600 cm^2^ for children)
ET	Exposure time (0.58 h day^−1^ for adults, 1 h day^−1^ for children)
CF	Unit conversion factor (0.001 L cm^−3^)
R_f_D	Oral reference dose (mg kg^−1^ day^−1^)^46^ Cr (0.003), Ni (0.029), As (0.003), Mo (0.005), Cd (0.0005), and Pb (0.0035)

#### Evaluation of non-carcinogenic hazards

The non-carcinogenic risk (N-CR) posed by individual PTEs through ingestion and skin contact was assessed by calculating the hazard quotient (HQ) through using Eqs. (3) & (4):

3 $$\:{HQ}_{ing}=\frac{{CDI}_{ing-ncr}}{{{R}_{f}D}_{ing}}$$

4 $$\:{HQ}_{der}=\frac{{CDI}_{der-ncr}}{{{R}_{f}D}_{der}}$$

where, *R*_*f*_*D*_*ing*_ and *R*_*f*_*D*_*der*_ represent reference doses for individual PTEs through ingestion and dermal routes, respectively as illustrated in Table 1. The dermal reference dose (*R*_*f*_*D*_*der*_) resulted from ingestion reference dose (*R*_*f*_*D*_*ing*_) using Eq. (5)^47,48^.

5 $$\:{{R}_{f}D}_{der}={{R}_{f}D}_{ing}\times\:GIAB\:$$

where, GIAB stands for the gastrointestinal absorption factor (Table 1) corresponding to the specific PTE under consideration. When the HQ is equal to or less than one, it suggests that adverse human health effects due to exposure to individual PTEs are improbable. However, when the HQ exceeds one, potential non-carcinogenic health concerns may arise. Additionally, the collective non-carcinogenic health risk resulting from exposure to all identified PTEs, referred to as the total hazard index (*HI*_*tot*_), was calculated using Eqs. (6, 7, and 8)^49,50^.

6 $$HI_{ing}=\Sigma HQ_{ing}$$

7 $$HI_{der}=\Sigma HQ_{der}$$

8 $$HI_{tot}=HI_{ing}+HI_{der}$$

The value of HI_tot_ greater than unity is deemed to present harmful non-carcinogenic health risk to human, whereas HI_tot_ lower than 1 suggests no non-carcinogenic risk from consuming various PTEs through potable water.

####  Evaluation of carcinogenic risk

The carcinogenic risk (CR) of a PTE can be estimated using Eqs. (9) and (10)^45^:

9 $$\:{CR}_{ing}={CDI}_{ing-ncr}\times\:{SF}_{ing}$$

10 $$\:{CR}_{der}={CDI}_{der-ncr}\times\:\:{SF}_{der}$$

where, *SF*_*ing*_ and *SF*_*der*_ are the carcinogenicity slope factors of corresponding target PTE for ingestion and dermal exposure pathways. These slope factors for oral intake were taken into account as 1.5, 0.5, 0.0061 and 0.0085 for Cr, As, Cd and Pb, respectively^43,51^. The CR associated with individual PTE is further categorized into different categories such as *CR* ≤ 10^−6^, 10^−6^ < *CR* ≤ 10^−4^, 10^−4^ < *CR* ≤ 10^−3^, 10^−3^ < *CR* ≤ 0.1, and *CR* > 0.1 indicates a very low, lower, moderate, higher and very higher cancerous risks, respectively due to exposure of target PTEs^52,53^. The slope factor for dermal exposure due to the target PTEs in different sub-population groups were obtained using Eq. (11)^44–54^. The combined carcinogenic risk (TCR) of all the analyzed PTEs was calculated using Eq. (12).

11 $$\:{SF}_{der}=\frac{{SF}_{ing}}{GIABS}$$

12 $$TCR=CR_{ing}+CR_{der}$$

### Soft computational methods for estimating water quality

In this research paper, radial basis function (RBF) and multilayer perceptron (MLP) neural network algorithms were combined for predicting the water pollution and health risk indices. For the RBF and MLP modeling, the eight indices were predicted using the measured PTEs as input variables. This has been described in Tables 2 and 3.

#### Radial basis function neural networks

Artificial neural networks (ANNs), including RBF models, have emerged as superior alternatives to multilinear regression in water quality simulation and prediction. A study by emphasized the capability of ANNs, such as RBFs, to offer valuable insights into the linearity and nonlinearity of input and output variables^55^. These networks exhibit higher compatibility and unpredictable features, making them effective substitutes for numerical indices and regression models^55–57^. Globally, researchers attest to the satisfactory results achieved by ANNs in modeling the intricate nonlinearities of water resources quality challenges^58^. Despite their advantages, it is crucial to acknowledge limitations in models like MLP and RBF^59,60^. RBF architecture, comprising input, hidden, and output layers interconnected by feedforward arcs, presents distinctive characteristics^61^. Learning occurs in two stages, first for the hidden layer based on input distribution and then for the output layer using supervised learning^58^. Unlike MLP, RBF’s hidden layer features radial functions, and parameter learning is unnecessary; linear weight modification and trial-and-error determine the appropriate number of neurons and spread numbers^62^. RBF’s structural simplicity and training efficiency find applications in pattern recognition, load forecasting, signal processing, and water quality prediction modeling^61^. The RBF stands out from back-propagation neural networks due to its guaranteed learning algorithm and single hidden layer, offering advantages in computational speed^61^. While back-propagated networks use stochastic approximation, RBF employs curve-fitting approximation with Gaussians, constructing local approximations for nonlinear input-output mapping^55,57,62^. The preference for localized approximation, supported by Walczak and Massart^63^, contributes to RBF’s growing popularity despite computational complexity and optimal parameterization challenges^61^. In a novel approach, this study employs RBF modeling for water pollution and health risk parameter simulation and prediction, utilizing the methodological commands outlined in Table 2.

**Table 2:** Outline of essential methodological commands crucial for crafting RBF models in this study.

Model parameter	Command/activity report
Input variables	*Pollution indices* – Cr, As, Cd, Pb, Ni, and Mo. *HI*_*ing*_ – Cr, As, Cd, Pb, Ni, and Mo. *CR*_*ing*_ – Cr, As, Cd, and Pb.
Output variable	*Pollution indices* – HPI, HEI, CI, and m-HPI. *HI*_*ing*_ – HI_ing (adult)_, HI_ing (child)_. *CR*_*ing*_ – CR_ing (adult)_, CR_ing (child)_.
ANN type	RBF
Rescaling of covariates	Normalized
Partitioning of dataset	Randomly assigned cases based on relative number of cases: *Pollution indices* – Training (75%), Testing (25%) *HI*_*ing*_ – Training (75%), Testing (25%) *CR*_*ing*_ – Training (80%), Testing (20%). The validity of all cases was 100%.
Number of hidden layers	One (1)
Hidden layer activation function	Softmax algorithm
Number of units	Automatically computed
Rescaling of scale dependent variables	Standardized
Output layer activation function	Identity

#### Multilayer perceptron neural networks

ANNs are a contemporary soft computational technology mimicking the human brain’s functionality, connecting artificial neurons to model datasets. Similar to RBF, research indicates that MLP effectively models the degree of linearity and nonlinearity between input and output variables^56^. The MLP, more extensively utilized than RBF, has been used to detect relationships between input and output factors in water pollution and quality modeling^56,62,64^. MLP’s reliable forecasting functions make it a popular choice for predicting water quality characteristics^53,56^. While RBF has its merits, MLP possesses distinct advantages in prediction studies, allowing flexibility with any nonlinear activation function, adaptive learning, deep learning capabilities, efficient learning of nonlinear models, direct derivation of decision functions from training datasets, and superior performance in classification problems compared to the RBFs^55^. The setup of the MLP models involved key steps, including (1) selecting the input data, (2) determining transfer function, (3) normalizing the dataset, (4) selecting the MLP architecture, (5) selecting the activation algorithms, (6) choosing the model performance criteria, and (7) selecting best MLP model^55,56,62^. In other words, optimizing the MLPs for water quality estimation entailed simultaneously training various networks with diverse number of neurons, activation functions, and training algorithms, with the optimal model selected at the end. Table 3 provides a summary of essential methodological information for creating the MLP models in this paper, emphasizing the intricate steps involved in achieving accurate predictions.

**Table 3:** Outline of essential methodological commands crucial for crafting MLP models in this study.

Model parameters	Command/activity report
Input variables	*Pollution indices* – Cr, As, Cd, Pb, Ni, and Mo. *HI*_*ing*_ – Cr, As, Cd, Pb, Ni, and Mo. *CR*_*ing*_ – Cr, As, Cd, and Pb.
Output variables	*Pollution indices* – HPI, HEI, CI, and m-HPI. *HI*_*ing*_ – HI_ing (adult)_, HI_ing (child)_. *CR*_*ing*_ – CR_ing (adult)_, CR_ing (child)_.
Hidden layer activation function	Hyperbolic tangent
ANN type	MLP
Rescaling of covariates	Normalized
Partitioning of dataset	Randomly assigned cases based on relative number of cases: *Pollution indices* – Training (70%), Testing (30%) *HI*_*ing*_ – Training (85%), Testing (15%) *CR*_*ing*_ – Training (70%), Testing (30%). The validity of all cases was 100%.
Number of hidden layers	One (1)
Output layer activation function	Hyperbolic tangent
Number of units	Automatically computed
Rescaling of scale dependent variables	Adjusted Normalized (Correction = 0.02)
Type of training	Batch
Optimization algorithm	Scaled conjugate gradient (SCG)

#### Performance verification of RBF and MLP models

Ensuring the practicality and reliability of artificial intelligence models in real-world scenarios involves their validation, considering that estimated values may not always align with the original dataset values^55^. Thus, model validation is crucial to assess the proximity of predictions to the originally measured data, serving as the foundation for selecting the most suitable models for implementation. Additionally, it aids in identifying optimal activation functions and optimization algorithms^56^. The verification of the RBF and MLP models’ performances in this research relied on determination coefficient (R^2^; Eq. 13), sum of square error (SOSE; Eq. 14), relative error (RE; Eq. 15), and residual error (Eq. 16) parity plots, providing a comprehensive evaluation framework for model effectiveness.

13 $$R^2=1-\frac{\sum_{i=1}^n\left({\mathrm X}_{\mathrm{predicted}\;\mathrm{value}}-{\mathrm X}_{\mathrm{measured}\;\mathrm{value}}\right)^2}{\sum_{i=1}^n\left({\mathrm X}_{\mathrm{predicted}\;\mathrm{value}}-{\mathrm X}_{\mathrm{average}\;\mathrm{value}}\right)^2}$$

14 $$SOSE=\sum_{i=1}^n\left({\mathrm X}_{\mathrm{measured}\;\mathrm{value}}-{\mathrm X}_{\mathrm{predicted}\;\mathrm{value}}\right)^2$$

15 $$RE=\frac{\left({\mathrm X}_{\mathrm{measured}\;\mathrm{value}}-{\mathrm X}_{\mathrm{predicted}\;\mathrm{value}}\right)}{{\mathrm X}_{\mathrm{measured}\;\mathrm{value}}}$$

16 $$\mathrm{Residual}\;\mathrm{error}={\mathrm X}_{\mathrm{measured}\;\mathrm{value}}-{\mathrm X}_{\mathrm{predicted}\;\mathrm{value}}$$

## Results and discussion

### Statistical analysis of physicochemical characteristics

Table 4 displays the statistical overview of physicochemical parameters, namely pH, electrical conductivity (EC), and total dissolved solids (TDS), observed in the examined groundwater sources. Table 5 presents the assessment of the suitability of the analyzed groundwater sources for drinking purposes, as determined by their EC and TDS values. The EC of analyzed samples varied between 160.88 and 578.29 µS cm^−1^ (AM = 373.19 µS cm^−1^), which lies within WHO acceptable limit of 1500 µS cm^[−1 65^.

**Table 4:** Statistical data regarding the observed physico-chemical parameters in groundwater samples from the Doon Valley in the Garhwal Himalayas (*N* = 40).

Water Quality Parameter	pH	EC	TDS
Unit	-	µS cm^−1^	mg L^−1^
Range	5.98 to 7.87	160.88 to 578.29	106.61 to 377.92
Median	7.22	435.89	276.77
A.M. ± S.D.	7.13 ± 0.49	373.19 ± 112.12	242.96 ± 71.82
G.M. ± S.D.	7.11 ± 0.49	353.14 ± 112.12	230.52 ± 71.82
MPL (BIS)	6.5–8.5	-	2,000
MPL (WHO)	-	1500	1000

AM- Arithmetic Mean, SD- Standard Deviation, GM- Geometric Mean, MPL- Maximum Permissible Limit, BIS- Bureau of Indian Standards, WHO- World Health Organization.

All the studied groundwater samples were deemed suitable for drinking according to their respective EC values (Table 5). Furthermore, TDS in all the water samples were also discovered to be within the acceptable limit of 500 mg L^−1^ recommended by the BIS^66^. The low EC and TDS values of groundwater make it suitable for drinking and other domestic uses. The water samples were observed to have pH ranging from 5.98 to 7.87 (AM = 7.13). The acidic nature of representative groundwater source could be linked to the natural carbonation occurring within the mineral water source^67^.

**Table 5:** Categorization of water types according to EC and TDS (*N* = 40).

S.*N*.	Parameter	Unit	Category	Suitability of Water	Samples (%)	Source
1.	EC	µS Cm^−1^	< 750	Desirable	100	^68^
			750 to1500	Permissible	Nil	
			1500 to 3000	Not permissible	Nil	
			> 3000	Hazardous	Nil	
2.	TDS	mg L^−1^	< 500	Desirable for drinking	100	^69^
			500 to 1,000	Permissible for drinking	Nil	
			1000 to 3000	Useful for irrigation	Nil	
			> 3000	Not suitable for drinking and irrigation	Nil	
3.	TDS	mg L^−1^	< 1000	Fresh water	100	^70^
			1,000–10,000	Brackish water	Nil	
			10,000–1,00,000	Saline water	Nil	
			> 1,00,000	Brine water	Nil	

It can, therefore, be concluded that observed physicochemical features of the examined water samples are in limits except for pH of a few samples. However, at larger perspective, this conclusion requires further study based on major anion and cation concentrations in groundwater sources of this region.

### Statistical analysis of PTEs distribution in Doon valley, Garhwal Himalaya

The levels of Cr, Ni, As, Mo, Cd, and Pb in the studied groundwater ranged as BDL to 0.388, BDL to 2.074, BDL to 0.573, BDL to 4.086, BDL to 0.404, and BDL to 3.937 µg L^−1^, respectively (Table 6). The average ranking of abundance dominance was Mo (1.066 ± 1.065 µg L^−1^) > Ni (0.744 ± 0.673 µg L^−1^) > Pb (0.337 ± 0.951 µg L^−1^) > As (0.186 ± 0.161 µg L^−1^) > Cr (0.180 ± 0.127 µg L^−1^) > Cd (0.026 ± 0.066 µg L^−1^). The detected levels of all the examined PTEs in the groundwater within the research area were notably lower than the maximum acceptable concentrations outlined by the World Health Organization^65^ and the Bureau of Indian Standards^66^.

**Table 6:** Variation of PTEs in groundwater samples from the Doon valley, Garhwal Himalaya (*N* = 40).

PTE concentration (µg L^−1^)	Acceptable limit		Min.	Max.	A.M.	Median	S.D.	Variance
	WHO^65^	BIS^66^						
Cr	50	50	BDL	0.388	0.180	0.172	0.127	0.014
Ni	70	20	BDL	2.074	0.744	0.561	0.673	0.230
As	10	10	BDL	0.573	0.186	0.130	0.161	0.026
Mo	70	70	BDL	4.086	1.066	0.659	1.065	1.132
Cd	3	3	BDL	0.404	0.026	0.011	0.066	0.004
Pb	10	10	BDL	3.937	0.337	0.026	0.951	0.399

Min- Minimum, Max- Maximum, BDL- Below Detection Limit.

The mean concentration of all the analyzed PTEs was also much lower than the maximum desirable limit (MDL), as recommended by the drinking water regulating agencies (Table 6). The frequency distribution of the PTEs concentrations across the study zone revealed that all the sampling stations had Cr, Ni, As, Mo, Cd, and Pb concentrations within their specified regulatory ranges, as depicted in Table 6.

### Evaluation of PTE pollution

The combined and thorough assessment of pollution levels for selected PTEs in the groundwater of the research area was conducted utilizing the HPI, m-HPI, HEI, and CI methodologies.

#### HPI

Estimated HPI values varied from **− 8.687** to **8.086** with mean value **− 0.265** during study period. The HPI is divided into three classifications according to their respective ranges: low (< 15), medium (15–30), and high (> 30)^71^. A critical threshold for the HPI is set at 100; water samples surpassing this value are deemed unsuitable for drinking purposes and should be disregarded^72^. All analyzed water samples (100%) found in low range therefore, were ranked as ‘excellent’ (Table 7). These results suggest non-contamination on PTEs content in that region.

**Table 7:** Appropriateness of analyzed water according to HPI, m-HPI, HEI and CI along with their % contribution in groundwater sources of study region (*N* = 40).

Pollution Indices	Classification	Suitability of Water	Category-wise contribution of locations	Percentage contribution	Critical Value	Ref.
HPI	< 25	Excellent	40	100	**100**	^72^
	26–50	Good	Nil	N.A.		
	51–75	Poor	Nil	N.A.		
	76–100	Very poor	Nil	N.A.		
	> 100	Unsuitable	Nil	N.A.		
m-HPI	−1 ≤ NI ≤ 0 and PI = 0	Excellent	40	100	NI ≤ 0 and PI > U_L_	^74^
	−1 < NI ≤ 0 and 0 < PI ≤ U_L_/2	Very good	Nil	N.A.		
	−1 < NI ≤ 0 and U_L_/2 < PI ≤ U_L_	Good	Nil	N.A.		
	NI ≤ 0 and PI > U_L_	Unacceptable	Nil	N.A.		
HEI	< 10	Low polluted	40	100	**20**	^73^
	10–20	Medium polluted	Nil	N.A.		
	> 20	Highly polluted	Nil	N.A.		
CI	< 1	Low polluted	40	100	**3**	^73,75^
	1–3	Moderate polluted	Nil	N.A.		
	> 3	Highly polluted	Nil	N.A.		

N.A. - Not Applicable, NI- Negative Index, PI- Positive Index, U_L_- Upper Limit.

#### m-HPI

HM pollution evaluated by m-HPI method showed no serious contamination like other methods (Table 7). According to the m-HPI water quality scale, 100% sampling locations of the study area were endured as ‘excellent’ (considerably safest), consequently, the water from those locations was deemed suitable for drinking and culinary use.

#### HEI

The estimated HEI values suggested that there was only minimal metal pollution across all stations in the study area, as shown in Table 7. HEI values ranged from 0.002 to 0.427 across all sampling sites, with a mean of 0.053 (± 0.074). The HEI is categorized into three classes: low (< 10), medium (10–20), and high (> 20)^73^. According to the HEI classification, all of the analyzed sampling stations (100%) exhibited ‘low’ metal pollution status. PTEs such as Cr, Ni, and Pb were not observed to be contributors for the HM pollution, accounting compositely for majority of the groundwater locations of the study area. However, due to the comparatively smaller number of PTEs and their lower prevalence in groundwater, the current study yielded lower HEI values.

#### CI

The calculated contamination factor (CF) values were recorded in the incidence of Ni (− 0.96278) < Pb (− 0.96421) < Mo (− 0.98477) < Cd (− 0.99091) < As (− 0.99629) < Cr (− 0.99641). However, CI values for the study area varied from − 5.958 (D38) to − 1.988 (D25) with an average of − 3.847 (± 0.945). According to the water quality classification criteria of CI, all studied sampling stations of study region exhibited low HM pollution (Table 7). Therefore, pollution levels were negligible at all stations within the study area.

Thus, based on the above analysis of estimated pollution indices it can be suggested that the groundwater of studied region is not contaminated w.r.t. different analyzed PTEs.

### Evaluation of health risk

The CDI of the identified PTEs through groundwater, either through direct ingestion or dermal contact, was calculated for adults and children (Tables 8 and 9). According to the mean CDI values, molybdenum (Mo) was found to be the most extensively consumed PTE on a daily basis through the direct ingestion of groundwater for both adults and children. Furthermore, Mo was observed to be the most absorbed element via dermal exposure for adults, whereas Cr for children. However, both adults and children at each site were observed to consume relatively lowest Cd via ingestion and dermal contact in comparison to remaining analyzed PTEs. It was also noted that the intake of the analyzed PTEs was greater through ingestion compared to dermal contact. The human health hazards due to continuous intake of PTEs via groundwater were assessed by computing non-carcinogenic and carcinogenic risks.

#### Non-carcinogenic health risks

The non-carcinogenic risks from the exposure of different target PTEs in groundwater in the studied locations can be appraised by comparing the calculated daily intake (CDI) with their respective reference R_f_D. The calculated values of the hazard quotients (HQs) for target PTEs reveal that exposure to ingestion for both adults and children is much higher than that for dermal contact (Tables 8 and 9). Thus, oral intake of water can be considered as a primary exposure route for PTEs in the public. Similar inferences have also been reported in a few other studies^76,77^. The health risks due to dermal exposure were almost negligible (≈ 0) for all analyzed PTEs. The mean HQ value of Mo was observed to be higher than that of other analyzed PTEs (Table 8). It concludes that potential non-carcinogenic health risks due to exposure of Mo are characterized as insignificant for the adults and children. The mean value of Cr was found to be highest among all analyzed PTEs through dermal contact for both population groups (Table 9). The estimated HQ values of Cr indicate that adults and children in the entire study area are not in danger to the Cr linked health implications.

**Table 8:** Non carcinogenic health risks of PTEs through drinking water via ingestion."Tables and its corresponding citations were renumbered. Please check if action taken is correct. Otherwise, kindly amend.""checked "

Element	Statistics	Concentration (µg L^−1^)	Adults		Children	
			CDI	HQ	CDI	HQ
Cr	Min	0.009	4.25E-07	1.42E-04	6.18E-07	2.06E-04
	Max	0.388	1.83E-05	6.11E-03	2.67E-05	8.88E-03
	AM	0.180	8.48E-06	2.83E-03	1.23E-05	4.11E-03
	SD	0.127	5.99E-06	2.00E-03	8.70E-06	2.90E-03
	GM	0.113	5.36E-06	1.79E-03	7.79E-06	2.60E-03
Ni	Min	0.011	5.20E-07	1.79E-05	7.56E-07	2.61E-05
	Max	2.074	9.80E-05	3.38E-03	1.42E-04	4.91E-03
	AM	0.744	3.52E-05	1.21E-03	5.11E-05	1.76E-03
	SD	0.673	3.18E-05	1.10E-03	4.62E-05	1.59E-03
	GM	0.347	1.64E-05	5.65E-04	2.38E-05	8.22E-04
As	Min	0.006	2.84E-07	9.45E-05	4.12E-07	1.37E-04
	Max	0.573	2.71E-05	9.03E-03	3.94E-05	1.31E-02
	AM	0.186	8.78E-06	2.93E-03	1.28E-05	4.25E-03
	SD	0.161	7.62E-06	2.54E-03	1.11E-05	3.69E-03
	GM	0.123	5.80E-06	1.93E-03	8.43E-06	2.81E-03
Mo	Min	0.001	4.73E-08	9.45E-06	6.87E-08	1.37E-05
	Max	4.086	1.93E-04	3.86E-02	2.81E-04	5.61E-02
	AM	1.066	5.04E-05	1.01E-02	7.33E-05	1.47E-02
	SD	1.065	5.04E-05	1.01E-02	7.32E-05	1.46E-02
	GM	0.525	2.48E-05	4.96E-03	3.61E-05	7.21E-03
Cd	Min	0.002	9.45E-08	1.89E-04	1.37E-07	2.75E-04
	Max	0.404	1.91E-05	3.82E-02	2.78E-05	5.55E-02
	AM	0.027	1.29E-06	2.58E-03	1.87E-06	3.75E-03
	SD	0.067	3.17E-06	6.34E-03	4.61E-06	9.21E-03
	GM	0.011	5.29E-07	1.06E-03	7.69E-07	1.54E-03
Pb	Min	0.005	2.36E-07	6.75E-05	3.43E-07	9.81E-05
	Max	3.937	1.86E-04	5.32E-02	2.70E-04	7.73E-02
	AM	0.358	1.69E-05	4.83E-03	2.46E-05	7.02E-03
	SD	0.978	4.62E-05	1.32E-02	6.72E-05	1.92E-02
	GM	0.043	2.02E-06	5.77E-04	2.94E-06	8.39E-04

**Table 9:** Non carcinogenic health risks due to different PTEs through water via dermal route.

Element	Statistics	Concentration (µg L^−1^)	Adults		Children	
			CDI	HQ	CDI	HQ
Cr	Min	0.009	1.29E-09	1.72E-05	2.04E-09	2.72E-05
	Max	0.388	5.55E-08	7.40E-04	8.80E-08	1.17E-03
	AM	0.180	2.57E-08	3.42E-04	4.07E-08	5.43E-04
	SD	0.127	1.81E-08	2.42E-04	2.87E-08	3.83E-04
	GM	0.113	1.62E-08	2.16E-04	2.57E-08	3.43E-04
Ni	Min	0.011	3.15E-10	5.42E-08	4.99E-10	8.60E-08
	Max	2.074	5.93E-08	1.02E-05	9.40E-08	1.62E-05
	AM	0.744	2.13E-08	3.67E-06	3.38E-08	5.82E-06
	SD	0.673	1.93E-08	3.32E-06	3.05E-08	5.26E-06
	GM	0.347	9.92E-09	1.71E-06	1.57E-08	2.71E-06
As	Min	0.006	8.58E-10	2.86E-07	1.36E-12	4.53E-10
	Max	0.573	8.19E-08	2.73E-05	1.30E-10	4.33E-08
	AM	0.186	2.66E-08	8.85E-06	4.21E-11	1.40E-08
	SD	0.161	2.30E-08	7.68E-06	3.65E-11	1.22E-08
	GM	0.123	1.76E-08	5.85E-06	2.78E-11	9.28E-09
Mo	Min	0.001	1.43E-10	2.86E-08	2.27E-13	4.53E-11
	Max	4.086	5.84E-07	1.17E-04	9.26E-10	1.85E-07
	AM	1.066	1.53E-07	3.05E-05	2.42E-10	4.83E-08
	SD	1.065	1.52E-07	3.05E-05	2.41E-10	4.83E-08
	GM	0.525	7.51E-08	1.50E-05	1.19E-10	2.38E-08
Cd	Min	0.002	2.86E-10	2.29E-05	4.53E-13	3.63E-08
	Max	0.404	5.78E-08	4.62E-03	9.16E-11	7.33E-06
	AM	0.027	3.90E-09	3.12E-04	6.18E-12	4.94E-07
	SD	0.067	9.59E-09	7.67E-04	1.52E-11	1.22E-06
	GM	0.011	1.60E-09	1.28E-04	2.54E-12	2.03E-07
Pb	Min	0.005	7.15E-10	2.04E-07	1.13E-12	3.24E-10
	Max	3.937	5.63E-07	1.61E-04	8.92E-10	2.55E-07
	AM	0.358	5.12E-08	1.46E-05	8.11E-11	2.32E-08
	SD	0.978	1.40E-07	4.00E-05	2.22E-10	6.33E-08
	GM	0.043	6.11E-09	1.75E-06	9.69E-12	2.77E-09

The estimated values of HI indicate that there are no potential non-carcinogenic health risks to the children and adults due to composite exposure to PTEs (Table 10). Therefore, it can be summed up that non-carcinogenic health risk of the target PTEs are negligible and can be ignored. However, children are more vulnerable to the non-carcinogenic health hazards associated with the identified PTEs compared to adults.

**Table 10:** Estimated values of total hazard index (HI) due to presence of PTEs in groundwater.

Site	Adults			Children		
	HI_(Ing)_	HI_(Der)_	HI_(Tot)_	HI_(Ing)_	HI_(Der)_	HI_(Tot)_
Min	0.001	0.000	0.001	0.001	0.000	0.001
Max	0.060	0.005	0.064	0.087	0.001	0.087
AM	0.018	0.000	0.019	0.026	0.000	0.027
SD	0.015	0.001	0.015	0.022	0.000	0.022
GM	0.013	0.000	0.013	0.019	0.000	0.019

#### Carcinogenic health risks assessment

Among the analyzed PTEs, Cr, As, Cd, and Pb are considered as carcinogen in the nature. Therefore, incremental lifetime cancer risks (ILCR) of these elements have been estimated for adults and children (Table 11). The estimated values of carcinogenic risk (CR) of Cr were observed to be lowest (**10**^**−6**^**<*CR*** **≤ 10**^**−4**^) through direct ingestion mode. The cancer mortality due to Cr exposure through potable water is reported in different studies^78,79^. Similarly, both adults and children in the study region can be considered to be very low susceptible (***CR*** **≤ 10**^**−6**^) to carcinogenic risk from Cr exposure, particularly via dermal contact to groundwater.

**Table 11:** Estimated carcinogenic risk of PTEs in groundwater sources.

Element	Statistics	Ingestion		Dermal	
		Adults	Children	Adults	Children
Cr	Min	6.38E-07	9.27E-07	1.93E-09	3.06E-09
	Max	2.75E-05	4.00E-05	8.32E-08	1.32E-07
	AM	1.27E-05	1.85E-05	3.85E-08	6.10E-08
	SD	8.98E-06	1.31E-05	2.72E-08	4.31E-08
	GM	8.04E-06	1.17E-05	2.43E-08	3.85E-08
As	Min	1.42E-07	2.06E-07	4.29E-10	6.80E-13
	Max	1.35E-05	1.97E-05	4.10E-08	6.49E-11
	AM	4.39E-06	6.38E-06	1.33E-08	2.11E-11
	SD	3.81E-06	5.53E-06	1.15E-08	1.83E-11
	GM	2.90E-06	4.22E-06	8.78E-09	1.39E-11
Cd	Min	5.77E-10	8.38E-10	1.74E-12	2.77E-15
	Max	1.16E-07	1.69E-07	3.52E-10	5.59E-13
	AM	7.86E-09	1.14E-08	2.38E-11	3.77E-14
	SD	1.93E-08	2.81E-08	5.85E-11	9.27E-14
	GM	3.23E-09	4.69E-09	9.77E-12	1.55E-14
Pb	Min	2.01E-09	2.92E-09	6.08E-12	9.63E-15
	Max	1.58E-06	2.30E-06	4.79E-09	7.59E-12
	AM	1.44E-07	2.09E-07	4.35E-10	6.90E-13
	SD	3.93E-07	5.71E-07	1.19E-09	1.88E-12
	GM	1.72E-08	2.50E-08	5.20E-11	8.23E-14

USEPA safe limits for Cr, As and Pb are ≤ 1 × 10^-6^.

The carcinogenic risks of As from the ingestion of groundwater are also observed lower (10^−6^ < *CR* ≤ 10^−4^) for both adults and children. The consumption of As has been observed to be associated with potential carcinogenic hazards in animals such as malignancies of the skin, bladder, liver, and lungs^80^. Looking at the estimated values of carcinogenic risk (*CR* ≤ 10^−6^), it can be inferred that adults and children of the study area are minutely susceptible to the carcinogenic risk of dermal contact to As in groundwater. Furthermore, the estimated risks from direct ingestion of Cd through potable groundwater was observed to be very low (*CR* ≤ 10^−6^) in the study area. Similarly, the carcinogenic risks from Cd exposure via dermal mode was also observed to be very (*CR* ≤ 10^−6^) to both adults and children. Therefore, it can be inferred that population of the study area is not affected by Cd related health disorders. The carcinogenic risks via oral route due to Pb exposure among adults and children were lower (10^−6^<*CR* ≤ 10^−4^) to very low (*CR* ≤ 10^−6^) in the entire region. However, health hazards owing to the Pb exposure through dermal absorption mode also indicate a very low (*CR* ≤ 10^−6^) cancerous risk among inhabitants. The combined risk of developing cancer from Cd exposure through ingestion and skin contact within both sub-populations of the study area is deemed very minimal, especially when compared to the risks associated with Cr, As, and Pb exposures. The total cancer risks (TCRs) resulting from combined exposure to carcinogenic PTEs via oral and dermal absorption modes indicate a lower cancer risk (10^−6^ < *CR* ≤ 10^−4^) for each sub-population groups by considering its highest and average values of the entire study region (Table 12).

**Table 12:** Estimated values of total carcinogenic health risk (TCR) due to presence of PTEs in groundwater.

Site	Adults			Children		
	CR(Ing)	CR(Der)	CR(Tot)	CR(Ing)	CR(Der)	CR(Tot)
Min	5.77E-10	1.74E-12	5.79E-10	8.38E-10	2.77E-15	8.38E-10
Max	3.11E-05	9.40E-08	3.12E-05	4.52E-05	1.32E-07	4.53E-05
AM	9.00E-06	2.73E-08	9.03E-06	1.31E-05	2.29E-08	1.31E-05
SD	9.03E-06	2.73E-08	9.06E-06	1.31E-05	3.95E-08	1.32E-05
GM	3.45E-06	1.04E-08	3.46E-06	5.02E-06	1.74E-10	5.02E-06

### Soft computational methods for estimating water quality

#### Radial basis function neural networks

**Figure 2:**
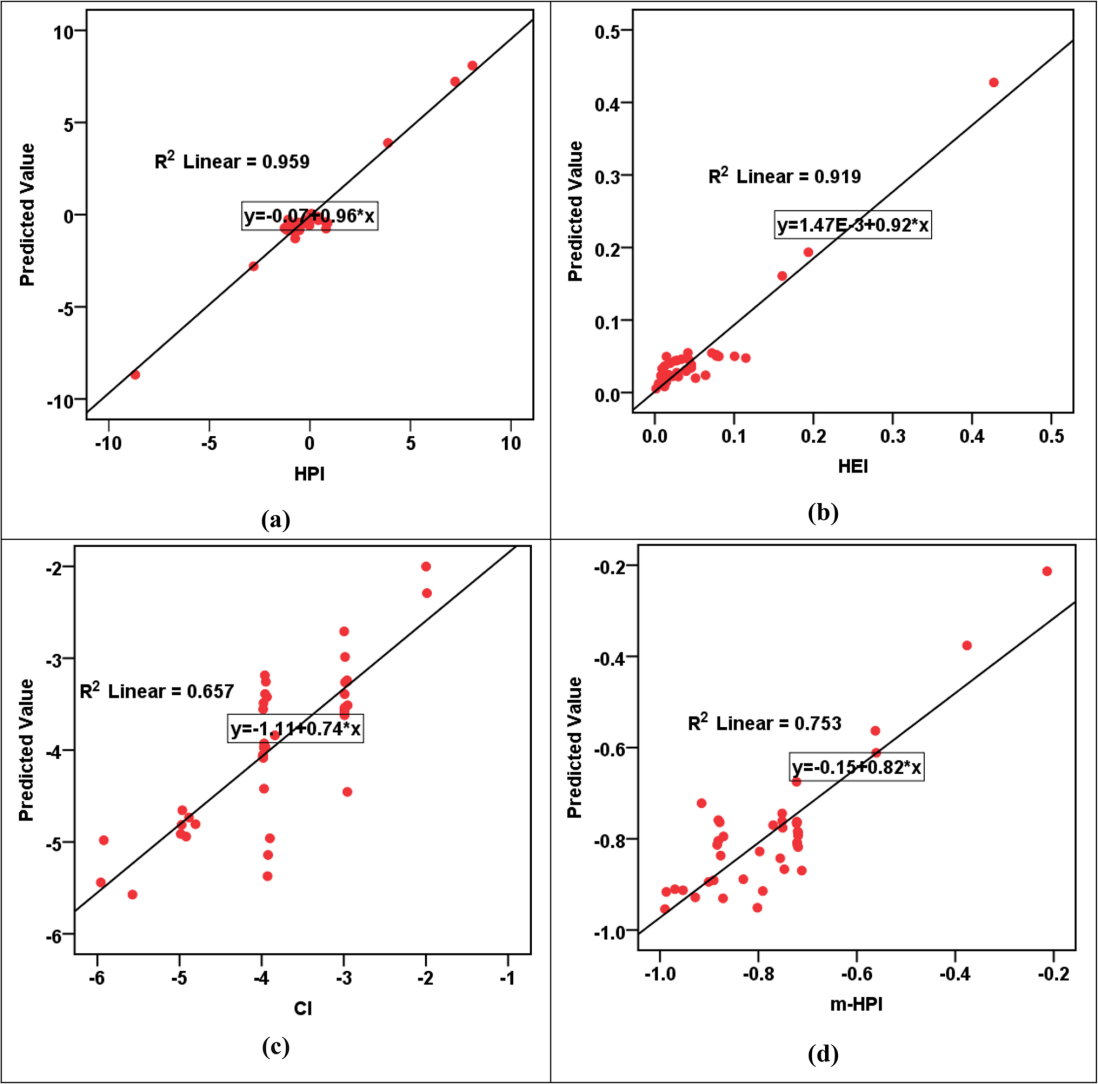
R^2^ parity plots for RBF prediction of (**a**) HPI, (**b**) HEI, (**c**) CI, and (**d**) m-HPI.

**Figure 3:**
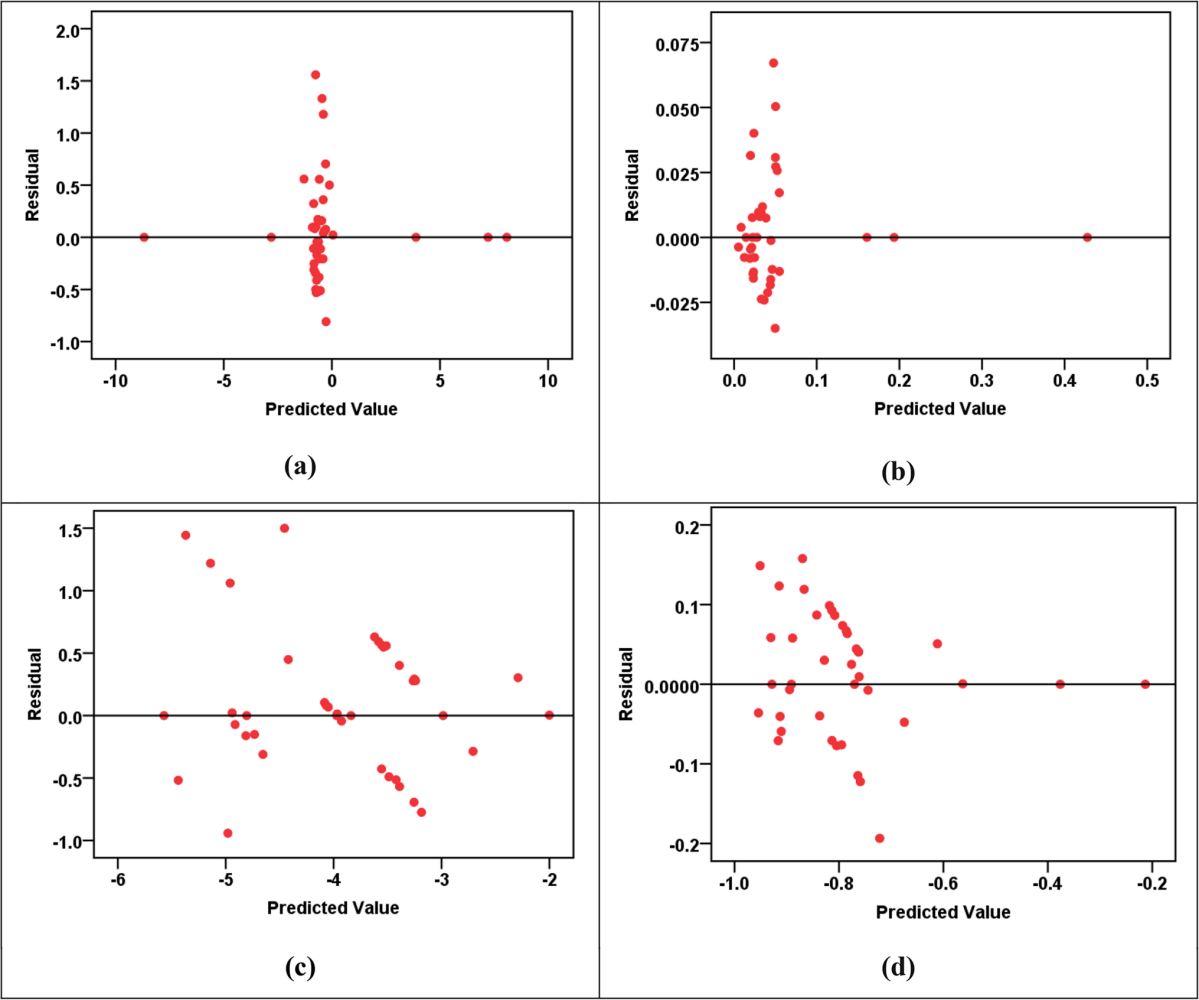
Residual error plots for RBF prediction of (**a**) HPI, (**b**) HEI, (**c**) CI, and (**d**) m-HPI.

**Figure 4:**
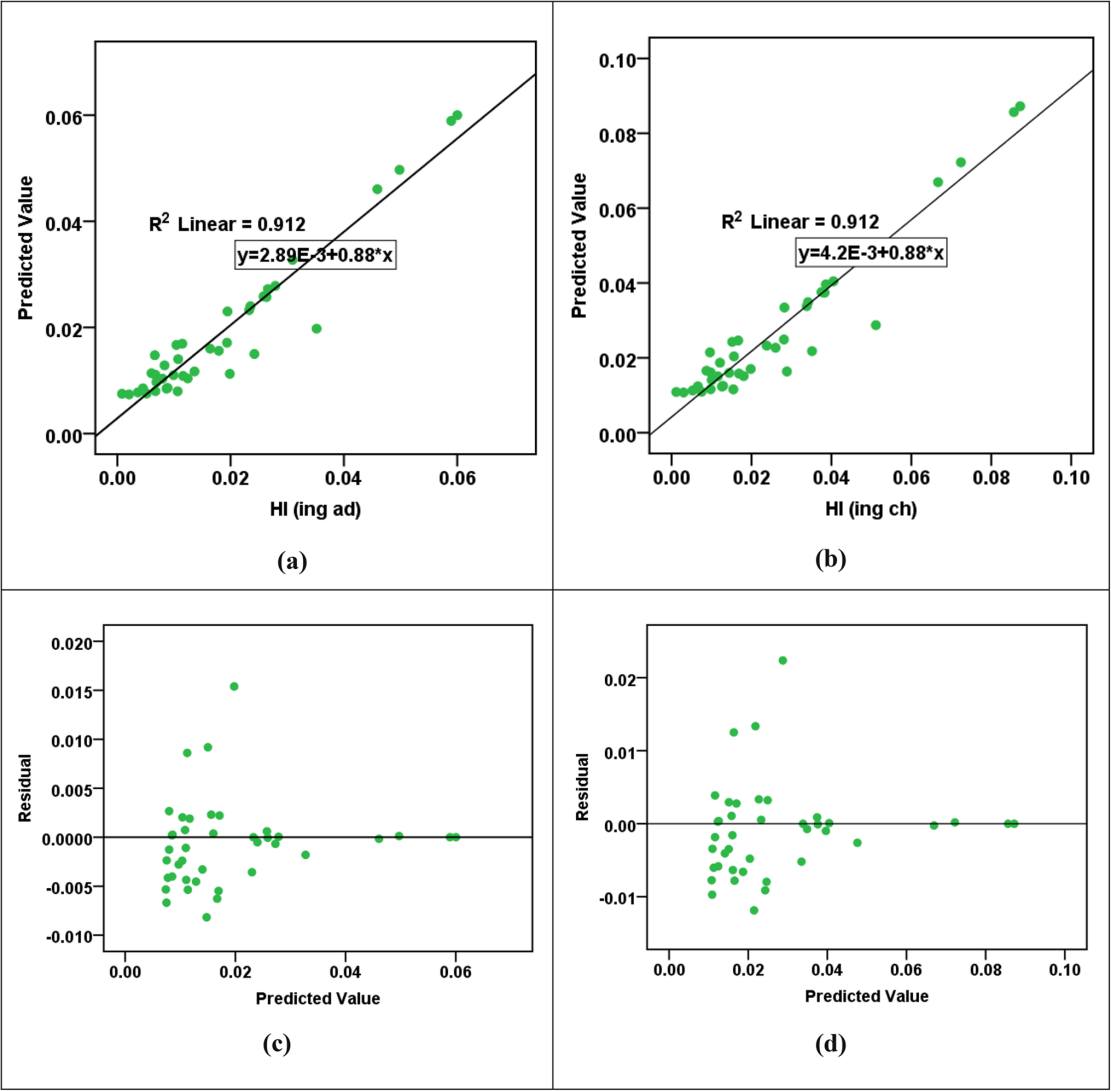
(**a** & **b**) R^2^ parity plots for RBF prediction of HI_ing (adult)_ and HI_ing (child)_. (**c** & **d**) Residual error plots for RBF prediction of HI_ing (adult)_ and HI_ing (child)_.

**Figure 5:**
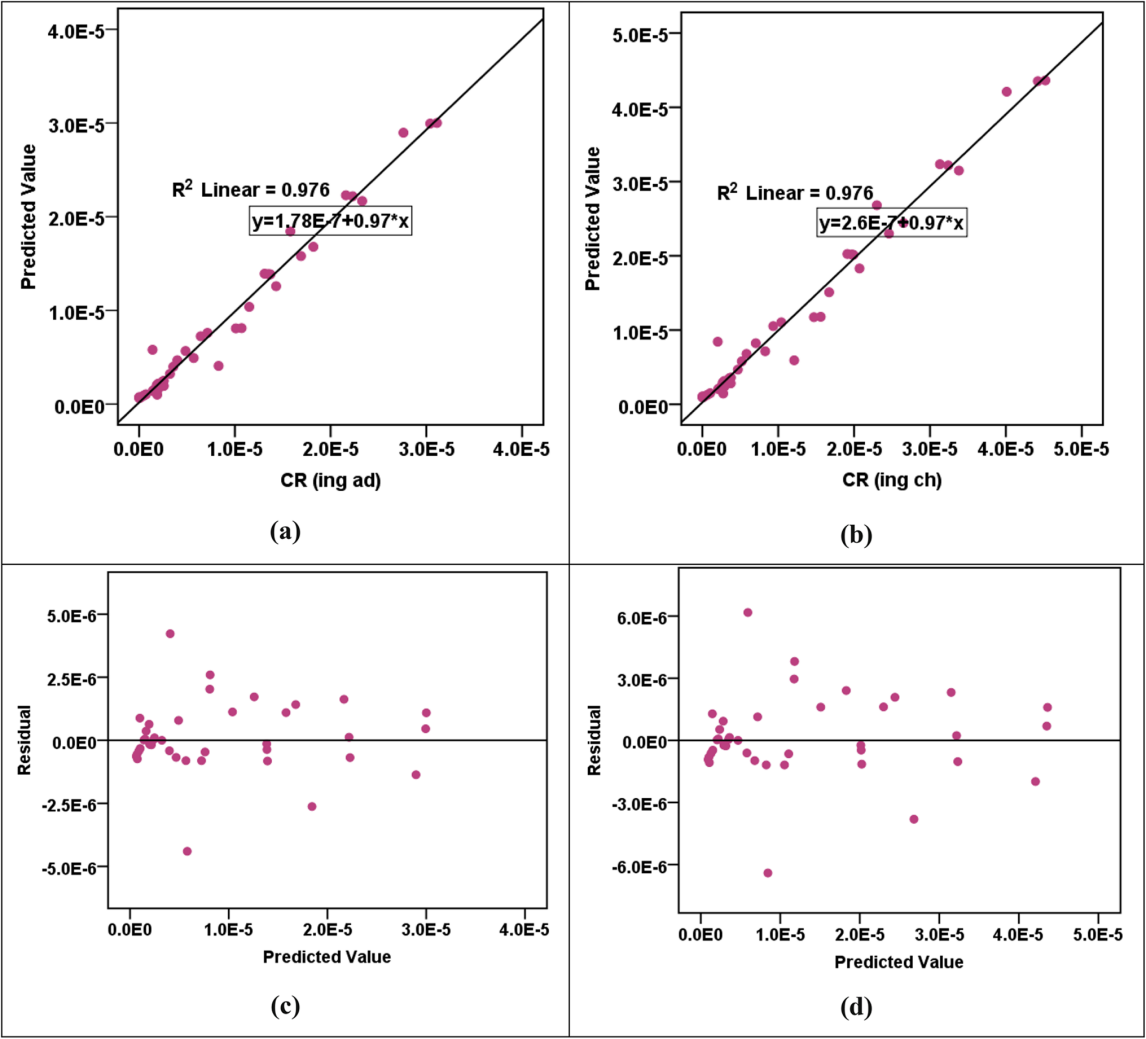
(**a** & **b**) R^2^ parity plots for RBF prediction of CR_ing (adult)_ and CR_ing (child)_. (**c** & **d**) Residual error plots for RBF prediction of CR_ing (adult)_ and CR_ing (child)_.

**Figure 6:**
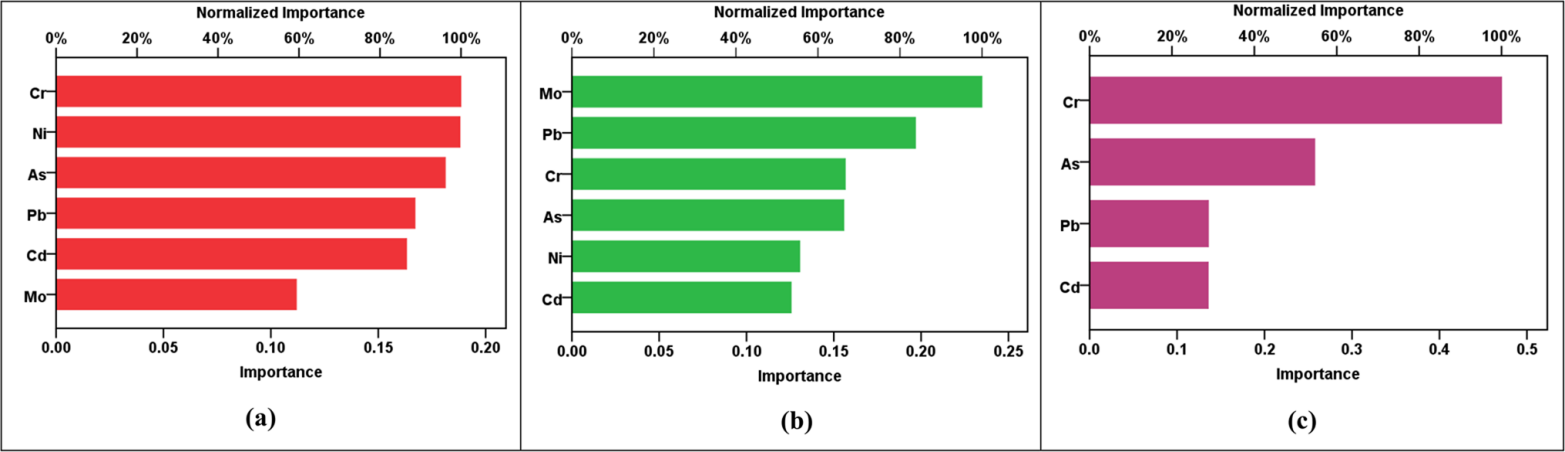
Sensitivity charts showing the importance of parameters for RBF prediction of (**a**) pollution indices, (**b**) hazard index (HI), and (**c**) cancer risk (CR).

The results of the RBF modeling for various parameters are shown in Table 13a. The R^2^ values indicated high levels of accuracy in predicting HPI, HEI, HI_ing (adult)_, HI_ing (child)_, CR_ing (adult)_, and CR_ing (child)_ parameters, with R^2^ values fluctuating from 0.912 to 0.976 in both training and testing phases. However, the CI and m-HPI predictions showed comparatively lower R^2^ values at 0.657 and 0.753, respectively. The SOSE values are consistently low across all parameters, reflecting the models’ precision. The RE values are much lower than the SOSE, indicating minimal discrepancies between predicted and actual values. Comparing the performance of RBF models across parameters, it is evident that HPI, HEI, HI_ing (adult)_, HI_ing (child)_, CR_ing (adult)_, and CR_ing (child)_ exhibit higher R^2^ values, suggesting superior predictive accuracy. In contrast, CI and m-HPI, while still reasonably accurate, show lower R^2^ values, indicating a slightly lower level of precision in predicting these parameters. The error and parity plots in Figs. 2, 3, 4, 5 and 6 further visualize the errors, demonstrating the varying degrees of alignment between predicted and actual values. The implications of these results are significant in the context of achieving the Sustainable Development Goals (SDGs) 3 and 6. The high accuracy in predicting health-related parameters (HI_ing_, CR_ing_) is crucial for monitoring and improving public health, aligning with SDG 3’s focus on ensuring healthy lives and promoting well-being. Additionally, the precision in predicting parameters related to water quality (HPI, HEI, CI, m-HPI) is essential for SDG 6, which aims to ensure access to water and sanitation for all, regardless of their ages, wealth and beliefs^27^. Thus, the modeling results provide a valuable tool for policymakers to make informed decisions, contributing to the successful implementation of SDGs 3 and 6.

**Table 13:** Performance measurement metrics for assessing the RBF and MLP models."Please check captured Table 13 if presented correctly.""Yes, it is fine. "

(a) RBF models						
	Parameter	R^2^	SOSE		RE	
			Training	Testing	Training	Testing
RBF – Pollution indices	HPI	0.959	6.163	6.058	0.023	0.802
	HEI	0.919	6.163	6.058	0.044	0.787
	CI	0.657	6.163	6.058	0.219	1.181
	m-HPI	0.753	6.163	6.058	0.139	2.348
RBF – HI	HI_ing (adult)_	0.912	2.499	0.686	0.086	0.273
	HI_ing (child)_	0.912	2.499	0.686	0.086	0.273
RBF – CR	CR_ing (adult)_	0.976	0.830	0.126	0.027	0.013
	CR_ing (child)_	0.976	0.830	0.126	0.027	0.013
(b) MLP models						
	Parameter	R^2^	SOSE		RE	
			Training	Testing	Training	Testing
MLP – Pollution indices	HPI	0.686	4.366	2.877	0.267	4.530
	HEI	0.954	4.366	2.877	0.050	0.253
	CI	0.437	4.366	2.877	0.450	0.936
	m-HPI	0.167	4.366	2.877	0.819	0.948
MLP – HI	HI_ing (adult)_	0.995	0.011	0.015	0.003	0.011
	HI_ing (child)_	0.994	0.011	0.015	0.005	0.011
MLP – CR	CR_ing (adult)_	0.887	3.204E-7	6.923E-7	0.063	0.336
	CR_ing (child)_	0.948	3.204E-7	6.923E-7	0.0.027	0.114

Figure 6 provides sensitivity charts revealing the significance of parameters in the RBF predictions for the pollution indices, hazard indices, and cancer risks. In Fig. 6a, the sensitivity analysis indicates that Cr, Ni, and As are the most influential elements in predicting the pollution indices (HPI, HEI, CI, and m-HPI). This suggests that the concentrations of Cr, Ni, and As play a pivotal role in determining the overall pollution levels in the area. Moving to Fig. 6b, the sensitivity analysis for hazard indices shows that Mo, Pb, and Cr have higher sensitivity in predicting the HIs for ingestion in both adult and child populations. Finally, Fig. 6c illustrates that Cr and As significantly influenced the RBF predictions of cancer risks for both adults and children. These observations bear crucial implications for future water monitoring and assessments. Firstly, understanding the key elements influencing the pollution indices allows for targeted monitoring of Cr, Ni, and As concentrations to effectively assess and mitigate environmental pollution. Secondly, the sensitivity of Mo, Pb, and Cr in predicting the health hazard indices reveals the importance of monitoring these elements for assessing health risks associated with ingesting the contaminated groundwater. Lastly, the influence of Cr and As on cancer risk predictions highlights the necessity of continuous monitoring and control measures for these elements, emphasizing their potential health impacts. These insights contribute to informed decision-making in environmental management, guiding future monitoring strategies and risk assessments to ensure the well-being of both adult and child populations.

#### Multilayer perceptron neural networks

**Figure 7:**
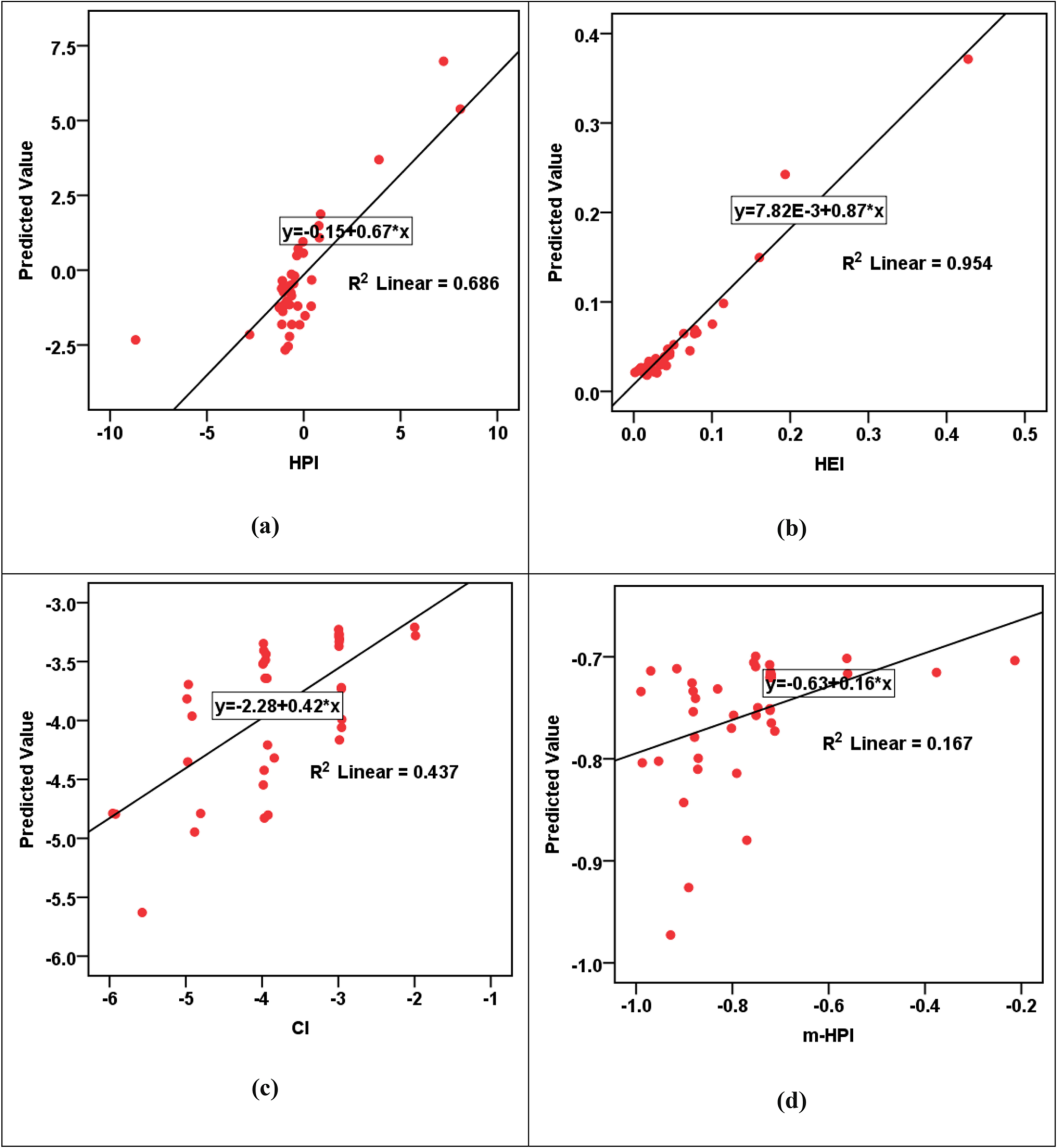
R^2^ parity plots for MLP prediction of (**a**) HPI, (**b**) HEI, (**c**) CI, and (**d**) m-HPI.

**Figure 8:**
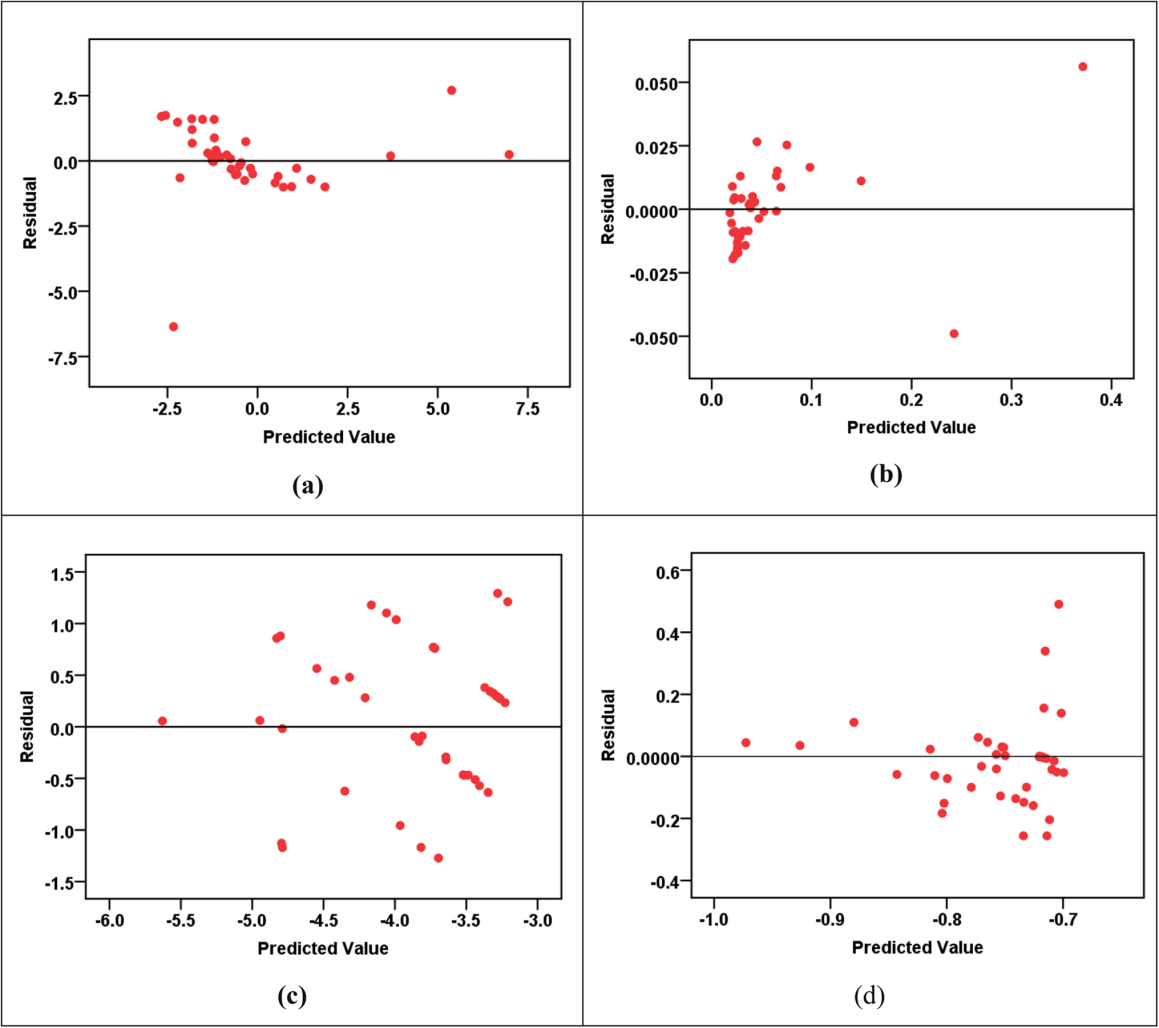
Residual error plots for MLP prediction of (**a**) HPI, (**b**) HEI, (**c**) CI, and (**d**) m-HPI.

**Figure 9:**
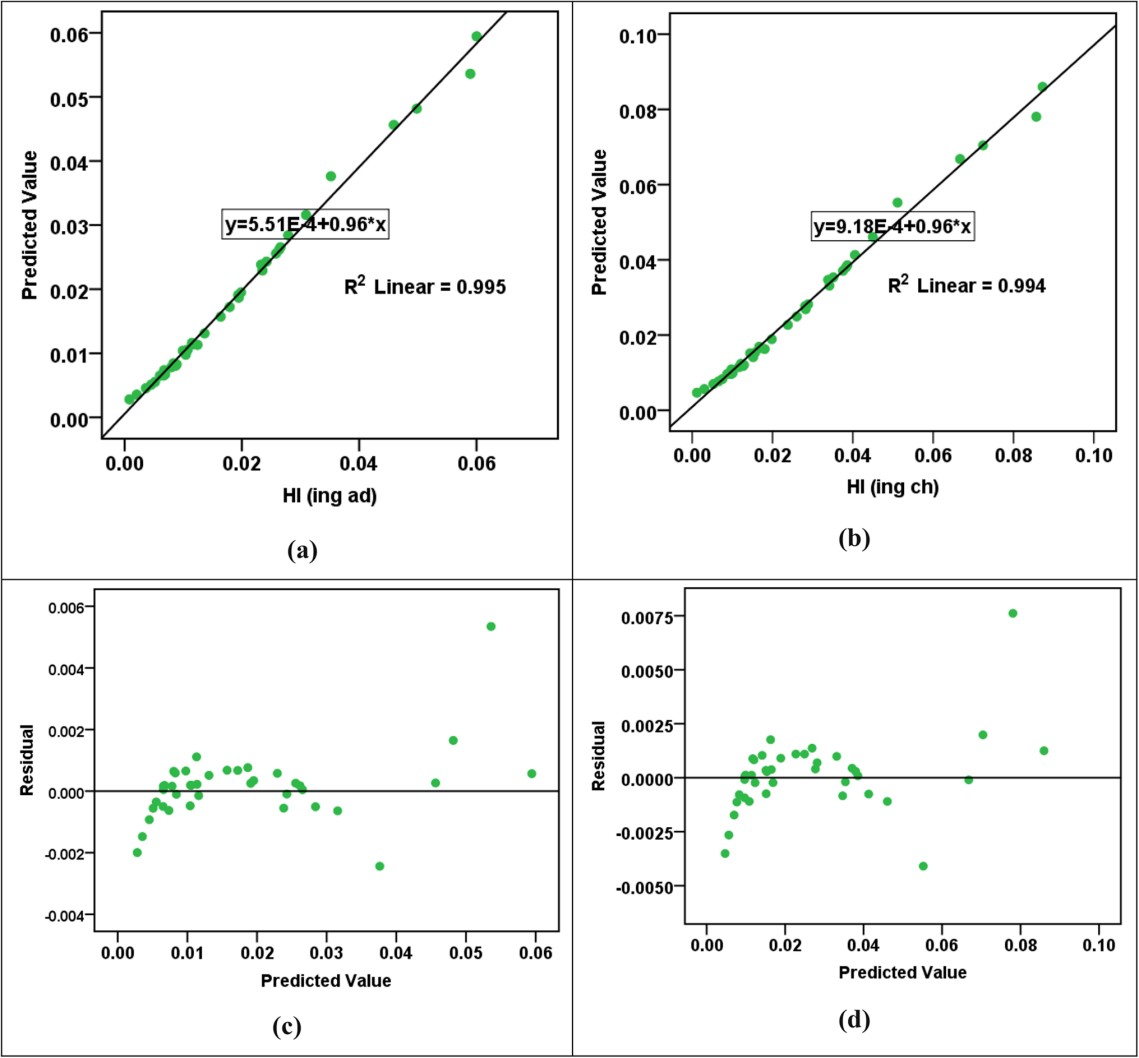
(**a** & **b**) R^2^ parity plots for MLP prediction of HI_ing (adult)_ and HI_ing (child)_. (**c** & **d**) Residual error plots for MLP prediction of HI_ing (adult)_ and HI_ing (child)_.

**Figure 10:**
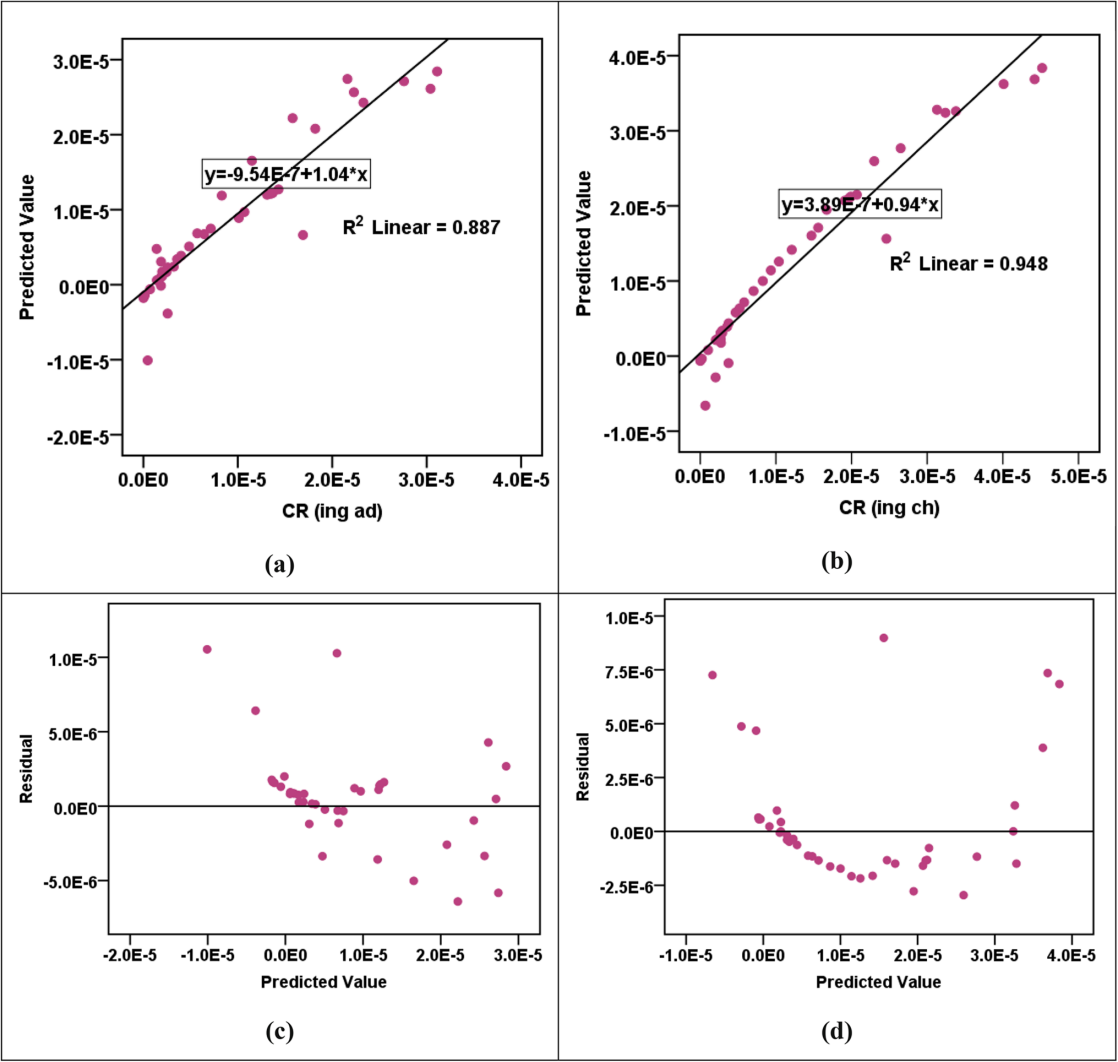
(**a** &** b**) R^2^ parity plots for MLP prediction of CR_ing (adult)_ and CR_ing (child)_ (**c** & **d**) Residual error plots for MLP prediction of CR_ing (adult)_ and CR_ing (child)_.

**Figure 11:**
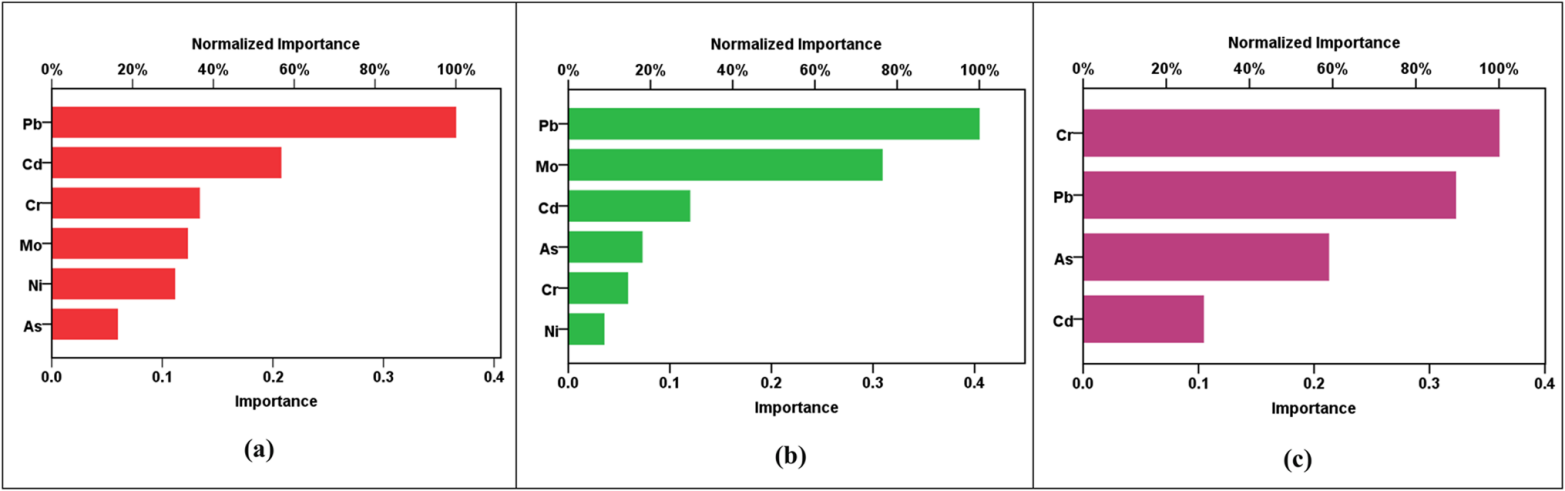
Sensitivity charts showing the importance of parameters for MLP prediction of (**a**) pollution indices, (**b**) hazard index (HI), and (**c**) cancer risk (CR).

The MLP modeling results, shown in Table 13b and Figs. 7, 8, 9, 10 and 11, unraveled a complex view of the predictive capabilities for the various parameters. Notably, the models exhibited a commendable proficiency in forecasting the hazard indices (HI_ing_ and CR_ing_ for both adults and children), showcasing elevated R^2^ values spanning from 0.887 to an impressive 0.995. This emphasizes the adeptness of the MLP models in capturing intricate relationships pertinent to the health risk assessments, especially in scenarios involving ingestion. Nevertheless, a discernable variation in precision arises as the models handled the prediction of the pollution indices (HPI, HEI, CI, m-HPI), with R^2^ values fluctuating between 0.167 and 0.954. Particularly noteworthy is the lowest accuracy in predicting m-HPI, hinting at inherent challenges in capturing this parameter accurately. A comparative analysis of the MLP and RBF performances unravels distinctive strengths within each paradigm. While the RBF models consistently outshined the MLP in forecasting the pollution indices, emphasized by superior R^2^ values across HPI, HEI, CI, and m-HPI parameters, the MLP was seen to have superior precision in predicting the hazard indices (HI_ing_ and CR_ing_). This becomes evident as MLP notches higher R^2^ values across all instances, signifying its prowess in delineating the health risks associated with the groundwater ingestion. The implications arising from the MLP modeling also offer a unique perspective on advancing the SDG 3 and SDG 6 objectives. The higher accuracy observed in predicting the hazard indices aligns well with the focus of SDG 3. Simultaneously, the noticed challenges in predicting the pollution indices affirms the necessity for continuous refinement of the MLP models. This emphasizes the long-term significance of continuous monitoring endeavors and assessments, which are very essential for refining the predictions. Figure 11 shows the sensitivity charts shedding light on the critical parameters influencing the MLP predictions for pollution indices, hazard indices, and cancer risks. In Fig. 11a, the sensitivity analysis points towards the significance of Pb and Cd as the most influential elements in predicting the HPI, HEI, CI, and m-HPI. From Fig. 11b, it was noticed that the sensitivity analysis for the HI predictions reveals that Pb and Mo exhibited higher sensitivity. Lastly, Fig. 11c elucidates that Cr and Pb significantly impacted the MLP predictions of cancer risks for both adults and children. These insights, just like those from the RBF modeling, underscore the need for continuous monitoring and control measures for these priority elements, as they have implications for public health. Looking forward, these observations could guide the allocation of resources toward targeted monitoring of Pb, Cd, Mo, and Cr to enhance the effectiveness of environmental management strategies and, consequently, advance the goals of SDGs 3 and 6.

#### More on the comparison of the RBF and MLP models

The comparison between the RBF and MLP models in this study provides valuable insights into their respective predictive performances across various parameters. Assessing the R^2^, SOSE, and RE values, as well as examining residual error plots, illuminates the strengths and limitations of each modeling approach. This is consistent with the approach adopted by previous researchers^81^. As cited earlier, in terms of R^2^ values, RBF consistently demonstrated higher accuracy in predicting the pollution indices, based on R^2^. The MLP, on the other hand, excelled better in predicting the hazard indices, with respect to R^2^. The differential nature of the results relates the importance of selecting modeling approaches tailored to specific outcomes. Examining the SOSE values further accentuated the robustness of the RBF models in predicting the pollution indices, while the MLP had the lowest errors associated with hazard indices prediction. The differential performance may be attributed to the inherent characteristics of each model. RBF, with its radial functions and local approximations, proved effective in capturing nonlinear relationships inherent in the pollution indices. In contrast, the MLP, with its flexible activation functions, adaptive learning, and capacity for deep learning, excelled in accurately assessing health risks associated with the groundwater ingestion. The residual error plots provided a visual representation of the model’s performance in predicting all indices. The RBF models displayed closer alignments between predicted and actual values for pollution indices, while MLP models showed greater precision in predicting the health indices. The distinctive patterns in these plots further emphasize the complex nature of the modeling results. The implications of this comparison are multidimensional. Understanding the strengths of each model allows practitioners to make informed decisions when selecting an approach based on the specific variables they aim to predict. The benefits extend to improved environmental management strategies, as accurate predictions are essential for devising targeted interventions. By leveraging the strengths of both the RBF and MLP models, stakeholders can enhance their ability to achieve the SDGs 3 and 6.

### Research implications and pathways for practical application of findings

The findings of this study have significant implications for public health, environmental management, and sustainable development in the Doon Valley region. The comprehensive analysis of PTEs in its groundwater provides crucial insights for addressing potential health epidemics and ensuring social welfare. While the current PTE levels are within acceptable limits, the study emphasizes the need/importance of continuous monitoring to prevent future contamination that could lead to serious health issues. The spatiotemporal analysis of risk assessment models developed in this research offer valuable tools for public health officials to identify and prioritize high-risk areas for intervention. These models can be adapted to create early warning systems for PTE contamination; thus, enabling proactive measures to safeguard the public health. Furthermore, the findings have implications for climate change adaptation and water resource management. As climate change may exacerbate water scarcity and alter the groundwater dynamics, understanding the current PTE distribution and potential risks is crucial for developing resilient water management strategies. The research provides a foundation for policymakers to integrate PTE monitoring into broader climate action plans. This would ensure that water quality remains a key consideration in adaptation efforts.

The results offer actionable insights for policymakers and environmental agencies to enhance groundwater management in the Doon Valley. By leveraging the analysis of PTE distribution, the authorities in charge of water resources can implement focused monitoring programs, with special attention to areas with higher potential for contamination. This approach would allow for more efficient use of limited resources while maximizing the effectiveness of water quality management efforts. Environmental agencies can use the risk assessment models to establish threshold values for PTEs that trigger specific mitigation actions, such as treatment interventions or restrictions on water use. The local authorities can develop and implement management strategies tailored to address high-risk areas identified in the study. These strategies may include promoting alternative water sources, implementing stricter regulations on industrial and agricultural activities that could contribute to PTE contamination, and investing in water treatment infrastructure in vulnerable communities.

The implications of this research extend beyond the immediate study area. It offers valuable insights for regions that are facing similar groundwater quality challenges. The risk assessment models and analytical approaches developed in this study can be adapted and applied to other geographies, providing a framework for comprehensive groundwater quality assessment. This transferability is particularly relevant for regions with similar geological characteristics or facing comparable anthropogenic pressures on water resources. The study’s findings also highlight the interconnectedness of water quality, public health, and food security. By ensuring the safety of groundwater used for irrigation, the research contributes to efforts to maintain food security and agricultural sustainability. The methodologies employed in this study can be integrated into broader water resource management plans, to help balance the competing demands of agriculture, industry, and domestic use while safeguarding the public health and the environment. As global attention focuses on achieving the SDGs, particularly SDG 3 and SDG 6, this research provides a practical roadmap for aligning local water management practices with these global objectives. That would demonstrate how scientific research can directly inform and support sustainable development initiatives.

### Uncertainties, limitations and recommendations

This paper mainly focused on the appraisal of water pollution and prediction of health hazard assessment models for PTEs. The input parameters used for the assessment of risk parameters are taken from the previous literature. The input parameters such as daily water intake rate, exposure period, body weight, exposed skin area, and exposure time were not observed in the study area but are used as generalized values. Better results would be generated, if there are region-based values for these parameters. The carcinogenic risks for Ni and Mo could not be calculated due to unavailability of CSF values for these elements. Some other limitations observed are listed below. They should be included and resolved in future studies:

- Extensive groundwater quality monitoring for longer period to identify temporal variations could not be performed due to scarcity of funding. There is need to extend the monitoring period to capture long-term temporal variations in groundwater quality, as demonstrated in the XAJ-NEW project (Zhang et al. 2022). This would provide valuable insights into the impacts of climate change on hydrological processes and PTE concentrations in the study area.
- This study presents the results of only six PTEs analyzed in the study. However, there are a number of other PTEs which should also be analyzed for classification of respective groundwater source.
- Some important cations viz. Ca^2+^, Mg^2+^, Na^+^, K^+^ and anions such as F^-^, Cl^-^, CO₃²⁻, HCO_3_^-^, SO₄^2-^ should also be taken into account for future hydrogeochemical studies.
- The analysis of microbiological parameters, which has not been done in this study, would add a comprehensive picture to the water quality of the study area.
- Need to implement a comprehensive, multiscale, and nested hydrometeorological monitoring program similar to the Xin’anjiang nested experimental watershed (XAJ-NEW) described by Zhang et al.^82^. This approach could help in understanding the spatiotemporal scaling effects of hydrological processes, revealing mechanisms controlling runoff generation and partitioning, and monitoring the contaminant hydrology in the Garhwal Himalaya region.
- Need to utilize more advanced modeling techniques, such as the adaptive Generalized Finite Element Method with global-local enrichment (GFEMgl) as proposed by He et al.^83^, to improve the accuracy of contaminant transport predictions in heterogeneous media. This method could be particularly useful for capturing the movement and transformation of PTEs in the complex groundwater system.

## Conclusions

Based on the rigorous and comprehensive assessment of the water quality characteristics of analyzed groundwater samples in this study, the specific conclusions can be given as follows:

- The physicochemical parameters and PTEs concentrations of groundwater samples have not been observed to be affected to a large extent by anthropogenic and geogenic activities.
- The analysis of HPI, m-HPI, HEI, and CI reveal that all the analyzed samples belong either to ‘excellent’ or to ‘low polluted’ category, concluding that investigated groundwater sources are suitable for drinking and other domestic needs.
- The results of HHRA show that there is no risk to adults and children via ingestion and dermal pathways due to investigated PTEs in the groundwater of study area.
- The RBF-NN model applied for estimation and prediction of various pollution indices, and HHRA showed strong predictions as its coefficient of determination (R^2^) spanned from 0.912 to 0.976 with low modeling errors, except CI and m-HPI.
- Similarly, the MLP-NN modeling technique, with low modeling errors also showed strong prediction, as its *R*^2^ ranged between 0.887 and 0.995, except m-HPI, CI, and HPI parameters.
- Based on the SOSE values, it accentuated the robustness of the RBF model in prediction of pollution indices, while the MLP had lowest errors associated with hazard indices prediction.
- The predictive modeling of groundwater quality was improved by combining data-intelligent algorithms, proving that using a combined modeling method is superior to using a single model.
- The results of this research will aid policymakers and water experts in effectively monitoring, evaluating, and managing groundwater resources in the specified area with a focus on sustainability.

## Data Availability

The data generated and analyzed in this study are available from the corresponding author upon reasonable request.
